# Accuracy of maxillary positioning using computer-designed and manufactured occlusal splints or patient-specific implants in orthognathic surgery

**DOI:** 10.1007/s00784-023-05125-9

**Published:** 2023-06-29

**Authors:** Yoana Malenova, Florian Ortner, Paris Liokatis, Selgai Haidari, Matthias Tröltzsch, Florian Fegg, Katharina T. Obermeier, Jens T. Hartung, Tamara K. Kakoschke, Egon Burian, Sven Otto, Hisham Sabbagh, Florian A. Probst

**Affiliations:** 1 Department of Oral and Maxillofacial Surgery and Facial Plastic Surgery, University Hospital LMU Munich, Munich, Germany; 2 Center for Oral, Maxillofacial, and Facial Reconstructive Surgery, Ansbach, Germany; 3 Institute of Diagnostic and Interventional Radiology, Technical University of Munich, School of Medicine, Munich, Germany; 4 Department of Orthodontics and Dentofacial Orthopedics, University Hospital LMU Munich, Munich, Germany

**Keywords:** PSI, Patient-specific implants, Occlusal splint, Orthognathic Surgery, CAD/CAM

## Abstract

**Objective:**

To determine the accuracy of maxillary positioning using computer-designed and manufactured occlusal splints or patient-specific implants in orthognathic surgery.

**Material and Methods:**

A retrospective analysis of 28 patients that underwent virtually planned orthognathic surgery with maxillary Le Fort I osteotomy either using VSP-generated splints (*n* = 13) or patient-specific implants (PSI) (*n* = 15) was conducted. The accuracy and surgical outcome of both techniques were compared by superimposing preoperative surgical planning with postoperative CT scans and measurement of translational and rotational deviation for each patient.

**Results:**

The 3D global geometric deviation between the planned position and the postoperative outcome was 0.60 mm (95%-CI 0.46–0.74, range 0.32–1.11 mm) for patients with PSI and 0.86 mm (95%-CI 0.44–1.28, range 0.09–2.60 mm) for patients with surgical splints.

Postoperative differences for absolute and signed single linear deviations between planned and postoperative position were a little higher regarding the x-axis and pitch but lower regarding the y- and z-axis as well as yaw and roll for PSI compared to surgical splints.

There were no significant differences regarding global geometric deviation, absolute and signed linear deviations in the x-, y-, and z-axis, and rotations (yaw, pitch, and roll) between both groups.

**Conclusions:**

Regarding accuracy for positioning of maxillary segments after Le Fort I osteotomy in orthognathic surgery patient-specific implants and surgical splints provide equivalent high accuracy.

**Clinical relevance:**

Patient-specific implants for maxillary positioning and fixation facilitate the concept of splintless orthognathic surgery and can be reliably used in clinical routines.

## Introduction

In orthognathic surgery, preoperative clinical findings, 2D radiographs, plaster models, and consecutive manual model surgery have been the basis of treatment planning for many years. Model surgery in semi-adjustable articulators has been transferred into the operating theatre using interocclusal splints [1, 2]. However, conventional techniques are limited due to lack of control in the third dimension, inaccuracy of face-bow transfer or interocclusal splints [3, 4], and autorotation of the temporomandibular joint in the supine and anesthetized patient [5]. Especially in patients with strong occlusal tilt and asymmetric deformities positioning of the maxilla has been demanding [6]. Another difficulty in conventional treatment planning is the impact on soft tissue and smile line.

To improve accuracy, computer-aided design (CAD) and computer-aided manufacturing (CAM) have been applied increasingly within the past decade. Furthermore, virtual treatment planning offers the possibility to simulate postoperative soft tissue prediction using preoperative computed tomography (CT) scans [7, 8]. Initial approaches such as computer designed and manufactured splints were the first promising techniques using virtual 3D planning [9–12]. Other techniques use locating guides accompanied with pre-bent titanium plates on the basis of a resin model manufactured using laser sintering rapid prototyping that depicts the planned outcome [13]. Another method to improve positioning is intraoperative simulation-guided navigation [14]. Newer waferless techniques use cutting guides and patient-specific implants (PSI) without interocclusal reference [15–17].

In general, virtual treatment planning regarding CAD/CAM surgical splints, navigation, and soft tissue planning appear to be accurate and reproducible methods for orthognathic surgery [18]. Few studies indicate that positioning using customized cutting guides and PSI leads to higher clinical accuracy in orthognathic surgery [19–21].

Yet, the best and most accurate transfer of virtual planning into surgery is necessary so that the advantages of CAD/CAM in orthognathic surgery come into use. Therefore, the aim of this study was to determine the accuracy of maxillary positioning in relation to virtual treatment planning using CAD/CAM patient-specific implants compared with VSP-generated surgical splints.

## Material and methods

### Study design

The presented survey is a retrospective single-center cohort study. The institutional review board authorized the study, and informed consent was waived (Ethics Committee, Ludwig-Maximilians-University, Munich, Germany: Ref.-No. 21-0164). Study participants were selected from an electronic database at our hospital. The database consecutively included all patients who received orthognathic surgery in the Department of Oral and Maxillofacial Surgery and Facial Plastic Surgery, University Hospital, Munich, Germany between January 2015 and December 2020. The study follows the standards for reporting observational studies (STROBE guidelines) [22].

### Study population

The study population consisted of patients who underwent virtually planned orthognathic surgery with maxillary Le Fort I osteotomy using computer designed and either manufactured surgical splints or PSIs. The decision on virtually planned surgical intervention and manufacturing of a VSP-generated splint or PSI was made in the context of an individual case decision of the treating surgeons each with more than 10 years of experience in this subject. Only primary maxillary or bimaxillary osteotomies were included in the study (uniformly as maxilla-first surgery). A further inclusion criterion was the availability of a post-interventional high-resolution computed tomography (CT, isotropic resolution ≤ 1 mm) in the Department of Radiology, University Hospital, Munich, Germany within 2 weeks after surgery. The CT scans in all cases were performed before the postoperative orthodontic readjustment. Patients with underlying syndromic disease, cleft lip, palate, or secondary orthognathic surgery as well as patients younger than 18 years were excluded from the study.

### Clinical workflow

Patients with indications for combined orthodontic orthognathic surgical treatment first received orthodontic pretreatment. After completion of pretreatment preoperative preparation for each patient included clinical examination by maxillofacial surgeons, high-resolution CT scans (isotropic resolution 0.625 mm), production of plaster models, and occlusal scans of the corresponding dental arch. Resulting DICOM data from CT scans and STL data from occlusal scans were transferred to an industrial partner for subsequent virtual surgical planning (VSP) using ProPlan CMF software (Materialise®, Leuven, Belgium). At first, the preoperatively collected high-resolution CT scans (DICOM data) were imported and lined up in the natural head position with respect to the Frankfurt horizontal plane, facial midline, and bibupillary line [23]. Subsequently, the occlusal scans (STL data) were imported and combined with the CT scan using a semiautomatic fusing algorithm resulting in a model with high-resolution of the dental arch.

**Fig. 1:**
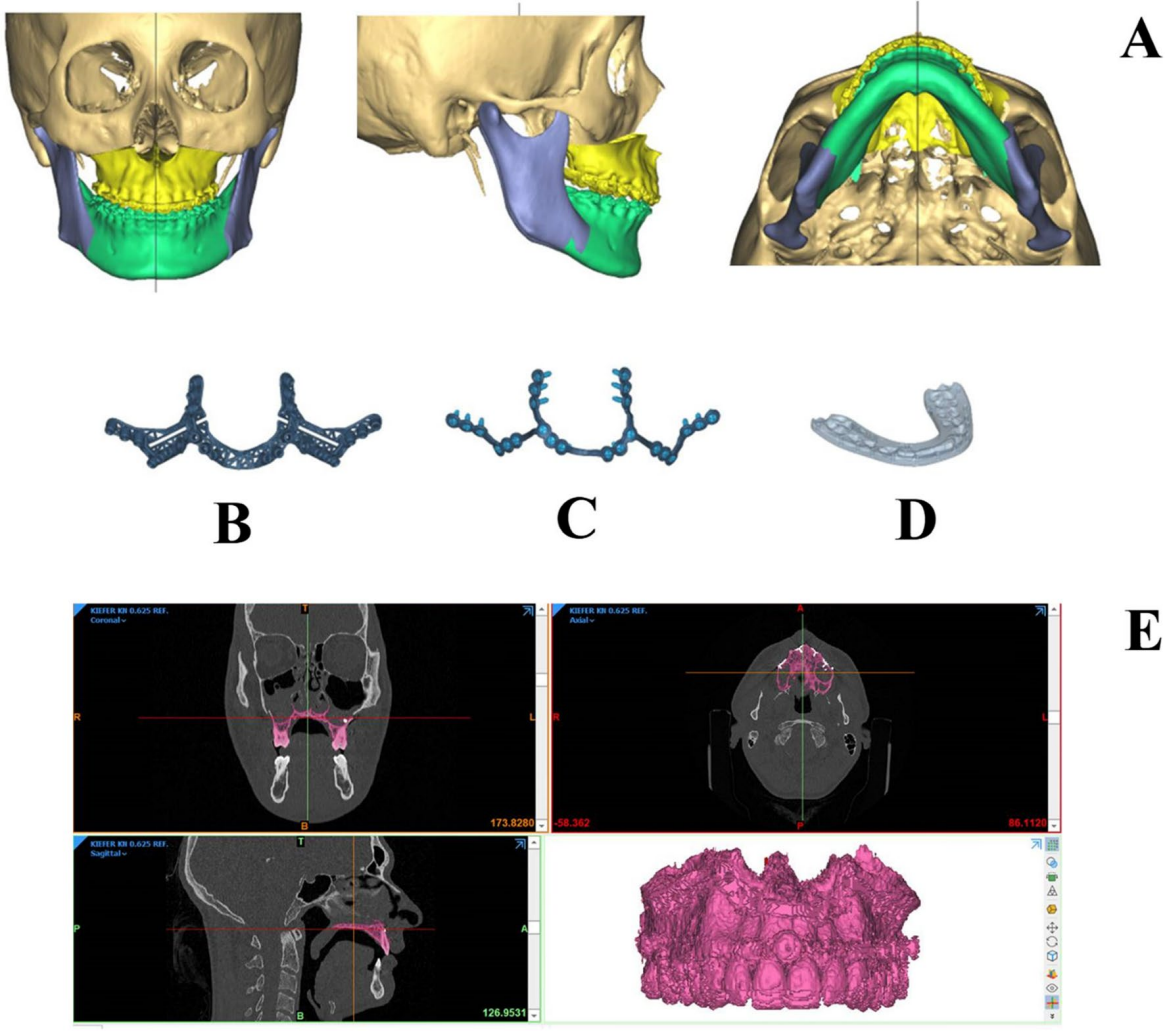
Planned postoperative position before bimaxillary orthognathic surgery with maxillary advancement and retropositioning of the lower jaw. Maxilla in yellow, mandibular body in green, and rami in blue (**A**), CAD/CAM cutting guide (**B**), patient-specific implants (PSI, **C**), and VSP generated surgical splints (**D**), segmentation of postoperative CT-scan using Mimics software (Materialise, Leuven, Belgium). The maxillary segment in purple (**E**)*

The following steps including cephalometric analysis, virtual Le Fort I, and BSS (Bilateral Sagittal Split) osteotomies for maxillary and mandibular movement respectively were performed with the corresponding analysis tools provided by the software. Based on the clinical findings and the virtual models, the segments of the upper and lower jaw were merged in final occlusion and the monoblock of the osteotomized maxilla and mandible then shifted into the final position in relation to the cranial base or the two articulated rami respectively (Fig. 1). Finally, an automated soft tissue simulation was performed using the same software (ProPlan CMF software, Materialise, Leuven, Belgium).

Positioning was checked regarding the position of the osteotomized maxilla and mandible (including the dental arches) in relation to the midface, skull base, center line, occlusal plane, and soft tissue.

This way, movement in all three dimensions of the osteotomized maxilla and mandible was encoded into the design and shape of either surgical splints or PSIs (Fig. 1B, C, and D).

After finishing the planning process and final approval by the surgeon the cutting guides, surgical splints as well as the PSIs went into computer-aided manufacturing (CAM) using resin based or selective laser melting (SLM), respectively. After sterilization, the operations were performed under general anesthesia by certified specialists for oral and maxillofacial surgery.

All cutting guides, as well as surgical splints or PSIs, were placed freehand, without the use of navigational systems or physical positioning guides. In bimaxillary surgery, maxillary repositioning was performed as the first step.

Postoperatively, high-resolution CT scans (isotropic resolution 0.625 mm) and routine ophthalmologic and follow-up clinical examinations were performed. Postoperative CT scans were performed as part of the clinical routine and before the start of any post-operative elastic treatment or otherwise orthodontic treatment.

### Data acquisition

The primary outcome variable was defined as the global geometric deviation between the virtually planned and the finally position of the maxilla determined by computed tomography in both groups.

First, postoperative CT scans were segmented using Mimics software (Materialise, Leuven, Belgium) differentiating soft tissue (HU < 300), bone tissue (HU 300–1500), and titanium (HU > 2000) (Fig. 1E).

**Fig. 2:**
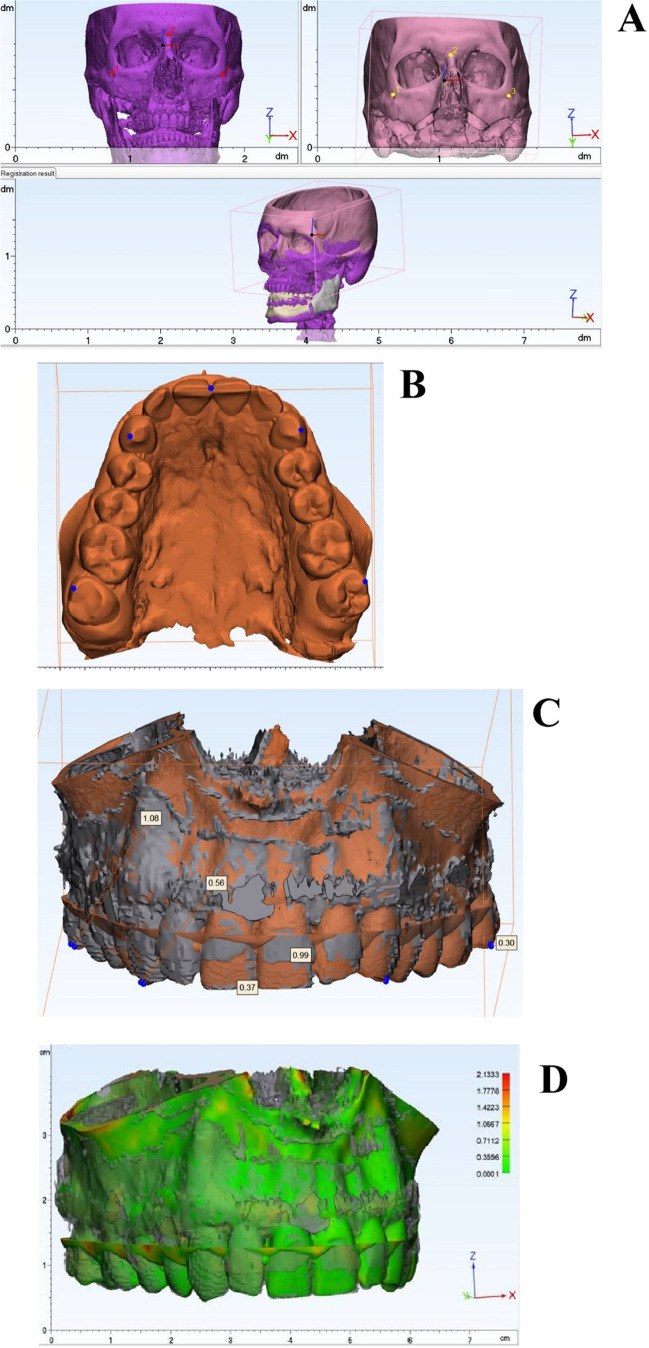
First alignment of planned (pink) and postoperative (purple) dataset using 3-matic software (Materialise, Leuven, Belgium) (**A**), selection of five measurement points: mesiobuccal cusps of the second molars, tips of the canines and mesial contact point of the incisors (**B**), measurement of direct distances between the respective five reference points of the maxillary teeth and subsequent evaluation of discrepancies in the spatial axes (**C**), color-coded heatmap visualizing areas with high (red) or low deviation (green) (**D**)

Data were exported as STL files (.stl) into 3-matic (Materialise, Leuven, Belgium), a dedicated CAD analyzing software. The corresponding STL files of the virtual treatment planning were provided by the industrial partner (Materialise, Leuven, Belgium) and imported into 3-matic as well. The non-osteotomized upper midfaces of the pre and postoperative datasets were aligned employing a semiautomatic superimposition algorithm. A 3-point alignment procedure (first alignment, Fig. 2A) followed by a semiautomatic superimposition algorithm (with 10 iterations) led to a matching of both datasets with an accuracy of approximately 30 µm (final alignment).

Subsequently, five measurement points were marked for each patient in the virtually planned and actual postoperative positions. For the best possible reproducible and precise measurement, five points with the greatest possible distance on the dental at the cusp tips of the maxillary teeth were chosen (the mesiobuccal cusps of the second molars, the tips of the canines, and the mesial contact point of the incisors) (Fig. 2B).

The geometric deviation between the virtually planned and the finally resulting position was compared by assessing differences in the entire bone surface and direct distances between the corresponding 5 selected reference points in spatial planes representing the primary outcome variables.

First 3D global geometric deviations were calculated. Therefore, the entire bone surface points of the virtually planned position were assigned to the respective closest points of the corresponding bone surface in the postoperative data set, and the respective Euclidean distances were measured.

Thereafter, direct distances between the respective five reference points of the maxillary teeth were measured and single linear deviations according to the spatial axes (x-, y-, z-axis) were evaluated with regard to absolute and signed linear deviations (Fig. 2C).

The x-axis is corresponding to transversal (lateral/medial), the y-axis is corresponding to sagittal (anterior/posterior), and the z-axis is corresponding to axial (cranial/caudal) movement. The planning model represented the starting point, and accordingly, the deviations of the postoperative position of the maxillary segment in the x-axis are defined as right/left. Differences to the right were defined as positive and those to the left as negative deviations.

For the assessment of rotational movements, angles in the corresponding planes were measured. Thus, for yaw angles in the x-y-plane, for pitch in the y–z-plane, and for a roll in the x-z-plane were evaluated.

Finally, color-coded heatmaps were generated to visualize areas with high or low deviation and therefore the distribution of geometric deviations (Fig. 2D).

### Statistics

Statistical analysis was performed using Excel (Microsoft, Redmond, USA) and SPSS 26 (SPSS Inc., Chicago, USA). Descriptive statistics were carried out for each study variable. Thus, means and standard deviations were calculated for global deviations between the bone segments, Euclidean distances, and absolute or signed distances in spatial axes for each measurement point in both groups as well as rotation angles.

For normally distributed data means were statistically compared by performing a student’s t-test. Normally distributed data was presented using mean ± standard deviation (SD).

Non-normally distributed data (according to Kolmogorov-Smirnov-Test und Shapiro-Wilk-Test) were statistically compared using the nonparametric Mann-Whitney U test. Non-normally distributed data were illustrated by depicting median and interquartile ranges.

Statistical significance was defined as *p* ≤ 0.05.

Intraclass correlation (ICC) assessed inter-rater agreement with respect to global deviations, Euclidean distances, and single linear deviations in the x-, y-, and z-axis of five corresponding measurement points as well as rotation angles.

## Results

The study included 28 patients that underwent virtually planned orthognathic surgery with maxillary Le Fort I osteotomy. In 15 patients (6 female, 9 male; average age: 27.6 years), maxillary positioning was conducted using PSI. In 13 patients (6 female, 7 male; average age: 27.5 years), surgical splints were used. In each group, there is one patient who only required correction of the upper jaw whereas the other patients underwent a bimaxillary osteotomy. In all cases, maxillary retrognathia was observed, and the choice of therapy in favor of the maxillary advancement was made accordingly.

Intraclass correlation (ICC) assessing inter-observer reliability of single linear deviations was 0.977 (PSI) and 0.918 (surgical splint).

### 3D global geometric deviation (in mm)

**Fig. 3:**
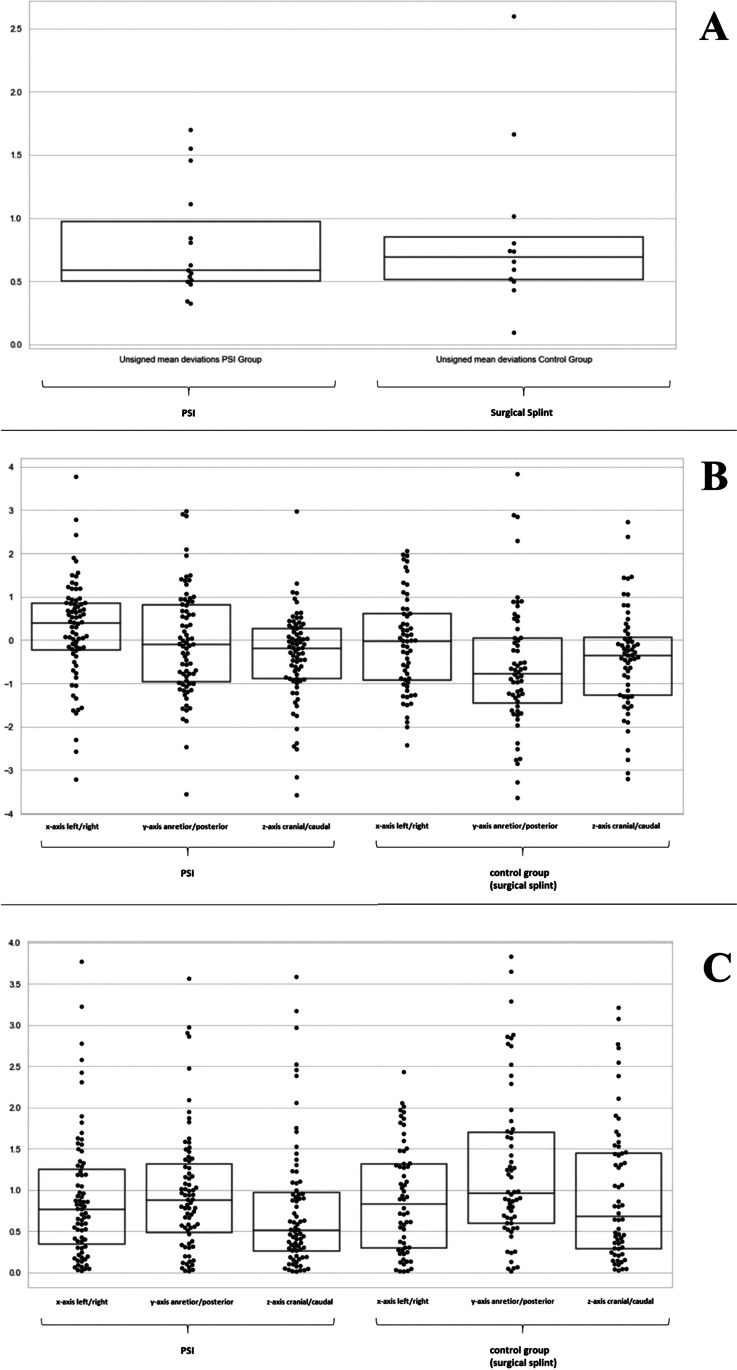
3D global geometric deviation (“mean surface distance”) between the planned position and the postoperative outcome was a little lower for patients with PSI (**A**), absolute linear deviations in the x-, y-, and z-axis for five corresponding reference points between the planned and the postoperative position for PSI group (on the left) and patients with VSP generated surgical splint (on the right) (**B**), signed linear deviations in the x-, y-, and z-axis for five corresponding reference points between the planned and the postoperative position for PSI group (on the left) and patients with VSP generated surgical splint (on the right) (**C**)

The 3D global geometric deviation between planned position and postoperative outcome referred to as “mean surface distance” was 0.60 mm (95%-CI 0.46–0.74, range 0.32–1.11 mm) for patients with PSI and 0.86 mm (95%-CI 0.44–1.28, range 0.09–2.60 mm) for patients with surgical splints. This difference was not statistically significant (level of significance *p* < 0.05). Compare Fig. 3A.

### Absolute linear deviations of five corresponding reference points according to the spatial axes (in mm)

Absolute linear deviations in the x-, y-, and z-axis for five corresponding reference points between the planned and the postoperative position were slightly higher regarding the x-axis but lower regarding the y- and z-axis for PSIs compared to surgical splints. The highest deviation was found in the y-axis for surgical splints. There were no statistically significant differences between the x-, y-, and z-axis comparing both groups (level of significance *p* < 0.05, Friedman test). Compare Table 1 and Fig. 3B.

**Table 1:** Absolute linear deviations in the x-, y-, and z-axis for five corresponding reference points between the planned and the postoperative position for PSI compared to surgical splints

	PSI	Surgical splint	*p*
x-axis (left/right)	0.90 mm (95%-CI 0.73–1.08, range 0.02–3.77 mm)	0.89 mm (95%-CI 0.72–1.06, range 0.01–2.43 mm)	1.00
y-axis (anterior/posterior)	0.97 mm (95%-CI 0.80–1.14, range 0.02–3.56 mm)	1.26 mm (95%-CI 1.01–1.50, range 0.01–3.83 mm)	1.00
z-axis (cranial/caudal)	0.77 mm (95%-CI 0.59–0.95, range 0.01–3.58 mm)	0.96 mm (95%-CI 0.74–1.18, range 0.02–3.21 mm)	0.25

### Signed linear deviations for five corresponding reference points (in mm):

Signed linear deviations in the x-, y-, and z-axis for five corresponding reference points between the planned and the postoperative position were higher regarding the x-axis (to the right) but lower regarding the y- and z-axis for PSIs compared to surgical splints. The highest deviation (posterior) was found in the y-axis for surgical splints. For patients with PSI maxillary segments by trend were positioned to the right. There were no statistically significant differences between the x-, y-, and z-axis comparing both groups (level of significance *p* < 0.05, Friedman test). Compare Table 2 and Fig. 3C.

**Table 2:** Signed linear deviations in the x-, y-, and z-axis for five corresponding reference points between the planned and the postoperative position for PSI compared to surgical splints

	PSI	Surgical splint	*p*
x-axis (right/left)	0.24 mm (95%-CI − 0.02–0.51, range 3.22–3.77 mm)	− 0.09 mm (95%-CI (− 0.38)–0.19, range (− 2.43)–2.05 mm)	0.42
y-axis (anterior/posterior)	− 0.06 mm (95%-CI (− 0.34)–0.23, range (-3.56)–2.97 mm)	− 0.63 mm (95%-CI (− 1.00)–(− 0.26), range (− 3.65)–3.83 mm)	0.22
z-axis (cranial/caudal)	− 0.37 mm (95%-CI (− 0.61)–(− 0.13), range (− 3.58)–2.97 mm)	− 0.44 mm (95%-CI (− 0.75)–(− 0.13), range (− 3.21)–2.72 mm)	1.00

### Rotations yaw, pitch, and roll (in degree)

**Fig. 4:**
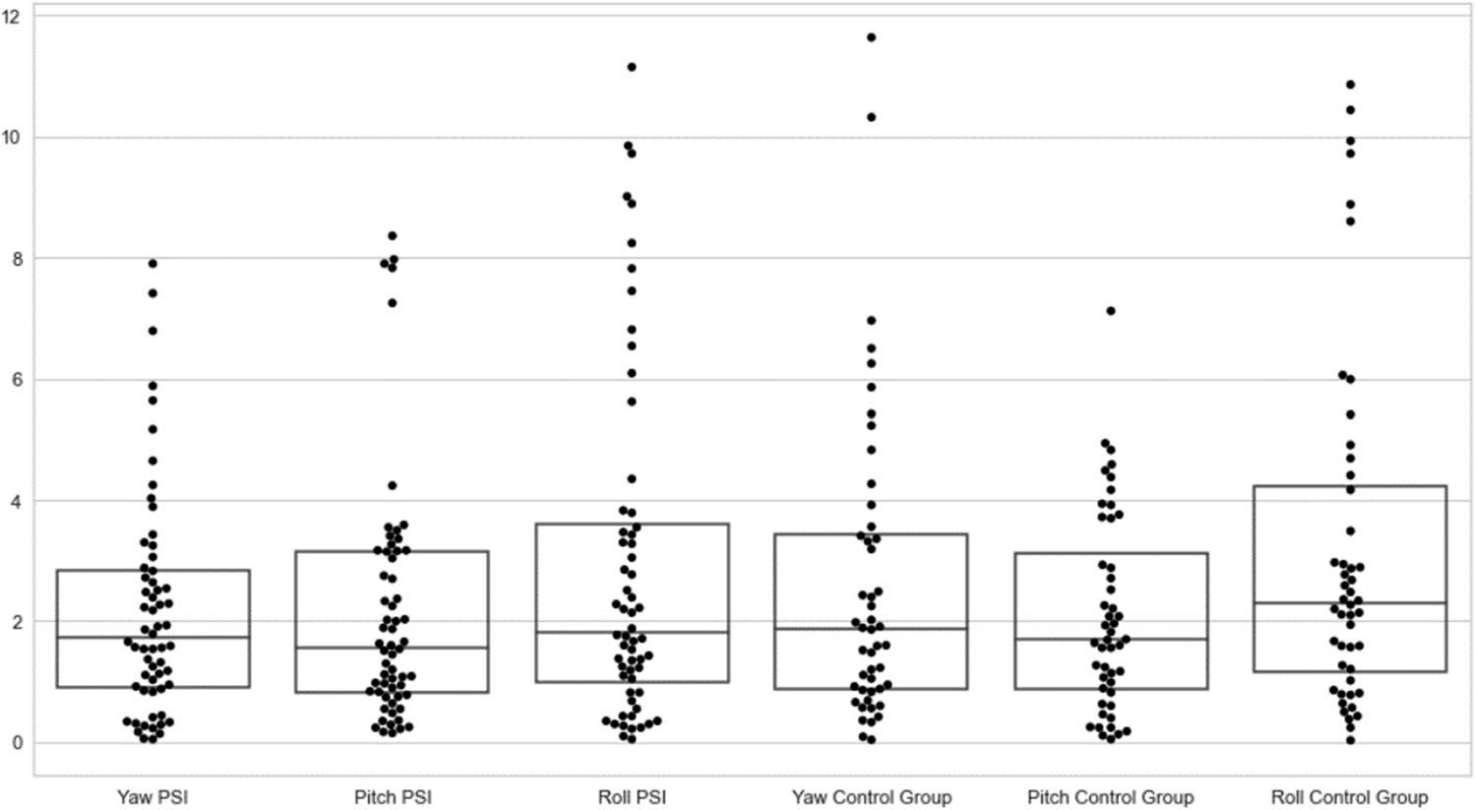
Differences in rotations pitch (transversal axis), yaw (longitudinal axis), and roll (sagittal axis) between the planned and the postoperative position for the PSI group (on the left) and patients with a surgical splint (on the right)

With respect to rotations pitch (transversal axis), yaw (longitudinal axis), and roll (sagittal axis) the planned and the postoperative position were a little higher regarding pitch but lower regarding yaw and roll for PSIs compared to surgical splints. There were no statistically significant differences between rotations comparing both groups (*p* < 0.05, Friedman test). Compare Table 3 and Fig. 4.

**Table 3:** Rotations yaw (longitudinal axis), pitch (transversal axis), and roll (sagittal axis) between the planned and the postoperative position for PSI compared to surgical splints

	PSI	Surgical splint	*p*
Yaw (longitudinal axis)	2.19 (95%-CI 1.71–2.67, range 0.05–7.90)	2.64 (95%-CI 1.90–3.38, range 0.04–11.64)	0.77
Pitch (transversal axis))	2.17 (95%-CI 1.64–2.70, range 0.15–8.36)	2.10 (95%-CI 1.62–2.57, range 0.05–7.12)	0.25
Roll (sagittal axis)	2.96 (95%-CI 2.22–3.71, range 0.05–11.15)	3.19 (95%-CI 2.34–4.04, range 0.03–10.86)	0.25

## Discussion

Particularly over the past decades, a lot of research groups and medical device manufacturers have made great efforts to improve 3D treatment planning and technical devices in order to improve accuracy and predictability in orthognathic surgery.

The first promising approaches included VSP-generated surgical splints, locating guides with pre-bent titanium plates on the basis of a resin model as well as intraoperative simulation-guided navigation [9–14].

Newer waferless techniques use cutting guides and patient-specific implants (PSI) without interocclusal reference [15–17], and some studies indicate that positioning using this technique leads to higher clinical accuracy in orthognathic surgery [19–21]. Therefore, the aim of this study was to determine the accuracy of maxillary positioning in relation to virtual treatment planning using either VSP-generated surgical splints or PSI.

Our results show comparable high accuracy of maxillary positioning using PSI or VSP-generated surgical splints with discrepancies less than 1 mm. In this study, there was no significant difference regarding any examined parameter for postoperative outcome after surgery between both techniques. Yet, deviations between the planned and postoperative position of the maxilla were lower with respect to 3D global geometric deviation, the y- and z-axis regarding for absolute and signed linear deviations in spatial axes as well as yaw and roll regarding rotations for PSIs compared to surgical splints.

Various studies have tried to analyze the accuracy of 3D virtually treatment planning of orthognathic surgery by different evaluating methods. Yet, there is no uniform measurement and statistical method available. The methods include measurement of linear and angular deviations between manually set landmarks, calculation of translational and rotational deviations with or without manually set reference points, color-coded heatmaps as well as intraclass coefficients [5, 9, 19–21, 24–34]. To achieve reliable results, this study combined these so far known evaluation methods. Besides artifacts in CT scans, every single method has its inaccuracy, such as imprecise manually set reference points or software-related inaccuracies. However, the different evaluation methods yielded comparable results in this study.

The main benefit of wafer-less surgery with CAD/CAM cutting guides and PSI is that positioning is conducted without interocclusal reference and therefore independent of the temporomandibular joint which should theoretically be more accurate for transferring the virtual plan into orthognathic surgery with maxillary Le Fort I osteotomy [19–21]. In our study, discrepancies tend to be a little lower for PSIs compared to surgical splints. Yet, VSP-generated surgical splints were a little more accurate regarding absolute and signed linear deviations in the x-axis as well as pitch in terms of rotational movements. For patients with PSI maxillary segments by trend were positioned to the right which might be caused by the operational perspective of the surgeon who is usually standing on the patient’s right side. The highest alterations were found for absolute and signed linear (posterior) deviations in the y-axis for surgical splints, which indicate an under-correction in maxillary advancement.

Given the small sample size of our study, missing significance in this analysis should not be interpreted as the definite absence of a real effect. By trend positioning of the maxillary segment was more accurate using PSI compared to VSP-generated splints.

Prospective randomized and controlled trials are necessary to finally assess which method is more accurate. However, our results show that both methods provide high clinical accuracy. The simultaneous use of PSI with VSP-generated surgical splints for maxillary positioning in orthognathic surgery might have a complementary effect.

## Conclusion

There were no statistically significant differences in the positioning of maxillary segments after Le Fort I osteotomy between patient-specific implants and surgical splints regarding 3D global geometric deviation, absolute and signed linear deviations in the x-, y-, and z-axis, and rotations (yaw, pitch, and roll) comparing both groups. Therefore, patient-specific implants and VSP-generated surgical splints provide comparable high accuracy for maxillary positioning in orthognathic surgery.

## Data Availability

The data that support the findings of this study are not publicly available due to confidentiality reasons. The anonymized statistical data is available from the corresponding author (YM) upon reasonable request.

## References

[1] Ellis E (1990). Accuracy of model surgery: evaluation of an old technique and introduction of a new one. J Oral Maxillofac Surg.

[2] Ritto FG, Schmitt ARM, Pimentel T, Canellas JV, Medeiros PJ (2018). Comparison of the accuracy of maxillary position between conventional model surgery and virtual surgical planning. Int J Oral Maxillofac Surg.

[3] Ellis E, Tharanon W, Gambrell K (1992). Accuracy of face-bow transfer: effect on surgical prediction and postsurgical result. J Oral Maxillofac Surg.

[4] Zizelmann C, Hammer B, Gellrich N-C, Schwestka-Polly R, Rana M, Bucher P (2012). An evaluation of face-bow transfer for the planning of orthognathic surgery. J Oral Maxillofac Surg.

[5] Sharifi A, Jones R, Ayoub A, Moos K, Walker F, Khambay B, McHugh S (2008). How accurate is model planning for orthognathic surgery?. Int J Oral Maxillofac Surg.

[6] Gateno J, Forrest KK, Camp B (2001) A comparison of 3 methods of face-bow transfer recording: implications for orthognathic surgery. J Oral Maxillofac Surg 59:635–40. discussion 640–1. 10.1053/joms.2001.23374 10.1053/joms.2001.23374 11381385

[7] Xia J, Ip HH, Samman N, Wong HT, Gateno J, Wang D (2001). Three-dimensional virtual-reality surgical planning and soft-tissue prediction for orthognathic surgery. IEEE Trans Inf Technol Biomed.

[8] Chabanas M, Marécaux C, Chouly F, Boutault F, Payan Y (2004). Evaluating soft tissue simulation in maxillofacial surgery using preoperative and postoperative CT scans. Int Congr Ser.

[9] Metzger MC, Hohlweg-Majert B, Schwarz U, Teschner M, Hammer B, Schmelzeisen R (2008). Manufacturing splints for orthognathic surgery using a three-dimensional printer. Oral Surg Oral Med Oral Pathol Oral Radiol Endod.

[10] Song K-G, Baek S-H (2009). Comparison of the accuracy of the three-dimensional virtual method and the conventional manual method for model surgery and intermediate wafer fabrication. Oral Surg Oral Med Oral Pathol Oral Radiol Endod.

[11] Hsu SS-P, Gateno J, Bell RB, Hirsch DL, Markiewicz MR, Teichgraeber JF (2013). Accuracy of a computer-aided surgical simulation protocol for orthognathic surgery: a prospective multicenter study. J Oral Maxillofac Surg.

[12] Schouman T, Rouch P, Imholz B, Fasel J, Courvoisier D, Scolozzi P (2015). Accuracy evaluation of CAD/CAM generated splints in orthognathic surgery: a cadaveric study. Head Face Med.

[13] Bai S, Shang H, Liu Y, Zhao J, Zhao Y (2012). Computer-aided design and computer-aided manufacturing locating guides accompanied with prebent titanium plates in orthognathic surgery. J Oral Maxillofac Surg.

[14] Mazzoni S, Badiali G, Lancellotti L, Babbi L, Bianchi A, Marchetti C (2010). Simulation-guided navigation: a new approach to improve intraoperative three-dimensional reproducibility during orthognathic surgery. J Craniofac Surg.

[15] Gander T, Bredell M, Eliades T, Rücker M, Essig H (2015). Splintless orthognathic surgery: a novel technique using patient-specific implants (PSI). J Craniomaxillofac Surg.

[16] Kraeima J, Jansma J, Schepers RH (2016). Splintless surgery: does patient-specific CAD-CAM osteosynthesis improve accuracy of Le Fort I osteotomy?. Br J Oral Maxillofac Surg.

[17] Suojanen J, Leikola J, Stoor P (2016). The use of patient-specific implants in orthognathic surgery: a series of 32 maxillary osteotomy patients. J Craniomaxillofac Surg.

[18] Stokbro K, Aagaard E, Torkov P, Bell RB, Thygesen T (2014). Virtual planning in orthognathic surgery. Int J Oral Maxillofac Surg.

[19] Heufelder M, Wilde F, Pietzka S, Mascha F, Winter K, Schramm A, Rana M (2017). Clinical accuracy of waferless maxillary positioning using customized surgical guides and patient-specific osteosynthesis in bimaxillary orthognathic surgery. J Craniomaxillofac Surg.

[20] Rückschloß T, Ristow O, Müller M, Kühle R, Zingler S, Engel M (2019). Accuracy of patient-specific implants and additive-manufactured surgical splints in orthognathic surgery - a three-dimensional retrospective study. J Craniomaxillofac Surg.

[21] Rückschloß T, Ristow O, Kühle R, Weichel F, Roser C, Aurin K (2020). Accuracy of laser-melted patient-specific implants in genioplasty - a three-dimensional retrospective study. J Craniomaxillofac Surg.

[22] von Elm E, Altman DG, Egger M, Pocock SJ, Gøtzsche PC, Vandenbroucke JP (2007). Strengthening the reporting of observational studies in epidemiology (STROBE) statement: guidelines for reporting observational studies. BMJ.

[23] Meiyappan N, Tamizharasi S, Senthilkumar KP, Janardhanan K (2015). Natural head position: an overview. J Pharm Bioallied Sci.

[24] Aboul-Hosn Centenero S, Hernández-Alfaro F (2012). 3D planning in orthognathic surgery: CAD/CAM surgical splints and prediction of the soft and hard tissues results - our experience in 16 cases. J Craniomaxillofac Surg.

[25] Badiali G, Ferrari V, Cutolo F, Freschi C, Caramella D, Bianchi A, Marchetti C (2014). Augmented reality as an aid in maxillofacial surgery: validation of a wearable system allowing maxillary repositioning. J Craniomaxillofac Surg.

[26] de Riu G, Meloni SM, Baj A, Corda A, Soma D, Tullio A (2014). Computer-assisted orthognathic surgery for correction of facial asymmetry: results of a randomised controlled clinical trial. Br J Oral Maxillofac Surg.

[27] Li B, Zhang L, Sun H, Yuan J, Shen SGF, Wang X (2013). A novel method of computer aided orthognathic surgery using individual CAD/CAM templates: a combination of osteotomy and repositioning guides. Br J Oral Maxillofac Surg.

[28] Shehab MF, Barakat AA, AbdElghany K, Mostafa Y, Baur DA (2013). A novel design of a computer-generated splint for vertical repositioning of the maxilla after Le Fort I osteotomy. Oral Surg Oral Med Oral Pathol Oral Radiol.

[29] Sun Y, Luebbers H-T, Agbaje JO, Schepers S, Vrielinck L, Lambrichts I, Politis C (2013). Accuracy of upper jaw positioning with intermediate splint fabrication after virtual planning in bimaxillary orthognathic surgery. J Craniofac Surg.

[30] Xia JJ, Gateno J, Teichgraeber JF, Christensen AM, Lasky RE, Lemoine JJ, Liebschner MAK (2007). Accuracy of the computer-aided surgical simulation (CASS) system in the treatment of patients with complex craniomaxillofacial deformity: a pilot study. J Oral Maxillofac Surg.

[31] Zinser MJ, Mischkowski RA, Sailer HF, Zöller JE (2012). Computer-assisted orthognathic surgery: feasibility study using multiple CAD/CAM surgical splints. Oral Surg Oral Med Oral Pathol Oral Radiol.

[32] Marchetti C, Bianchi A, Bassi M, Gori R, Lamberti C, Sarti A (2006) Mathematical modeling and numerical simulation in maxillo-facial virtual surgery (VISU). J Craniofac Surg 17:661–7. discussion 668. 10.1097/00001665-200607000-00009 10.1097/00001665-200607000-00009 16877910

[33] Hernández-Alfaro F, Guijarro-Martínez R (2013). New protocol for three-dimensional surgical planning and CAD/CAM splint generation in orthognathic surgery: an in vitro and in vivo study. Int J Oral Maxillofac Surg.

[34] Baan F, Liebregts J, Xi T, Schreurs R, de Koning M, Bergé S, Maal T (2016). A new 3D tool for assessing the accuracy of bimaxillary surgery: the orthognathic analyser. PLoS One.

